# Supplementary material for: Relating form to function in the hummingbird feeding apparatus
Source: PeerJ. 2017 Jun 8;5:e3449. doi: 10.7717/peerj.3449 (PMC5466813; doi:10.7717/peerj.3449)
Supplement: Supplemental Information 1 — Legends in the file [file peerj-05-3449-s001.docx]

**Supplementary Information –** Relating form to function in the hummingbird feeding apparatus


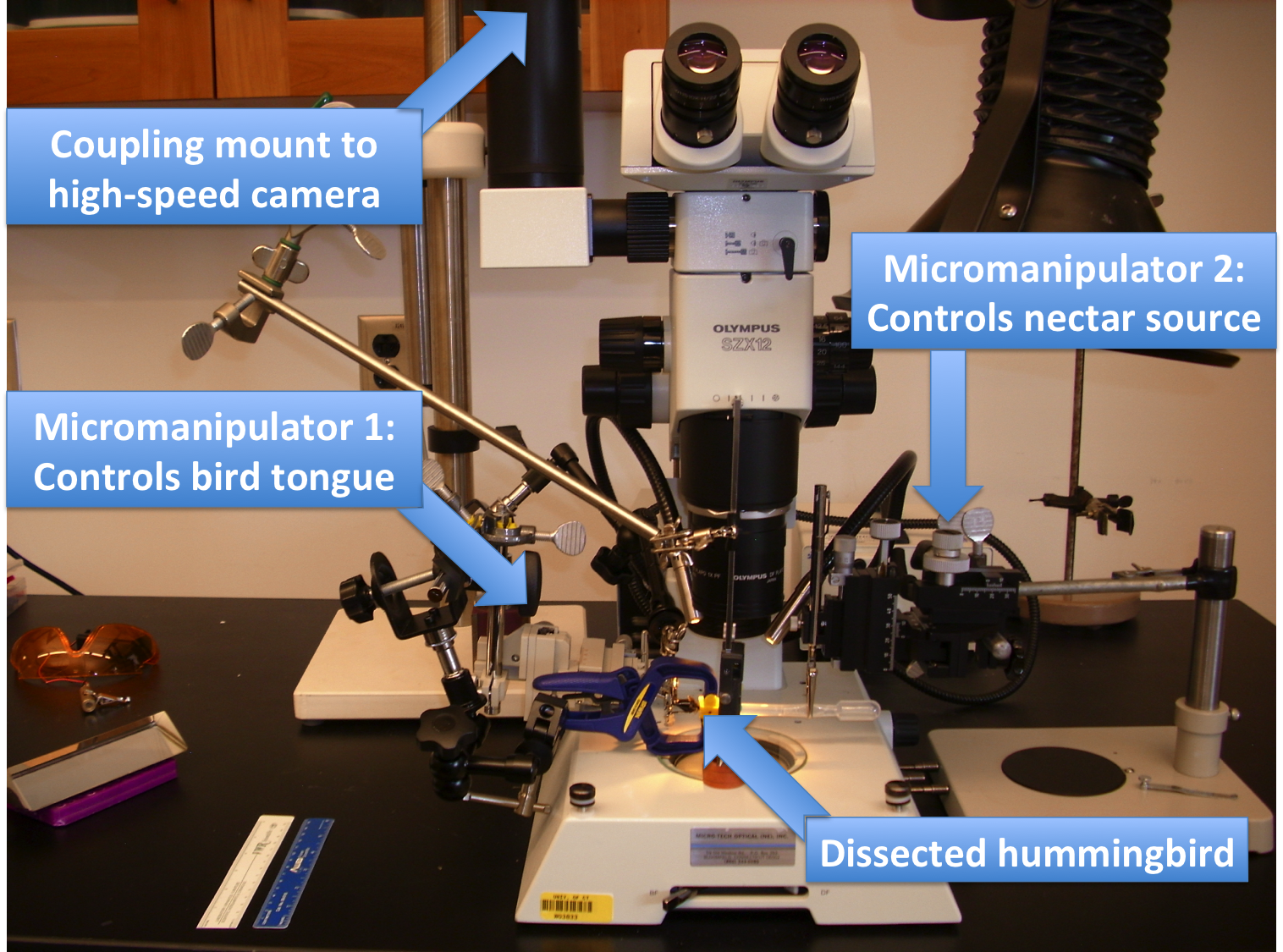


**Figure S1**. **Experimental setup to film *post-mortem* tongue fluid interactions.** Under a dissecting microscope (Olympus SZX-12), I build a control system to emulate reciprocating tongue movements and distance to the nectar surface using micromanipulators (Model FX-117, Electron Microscopy Sciences^©^). I coupled a high-speed camera (TroubleShooter HR at 1260 frames/s - 1280 x 512 pixels) to the dissecting microscope to capture the details of tongue fluid interactions.


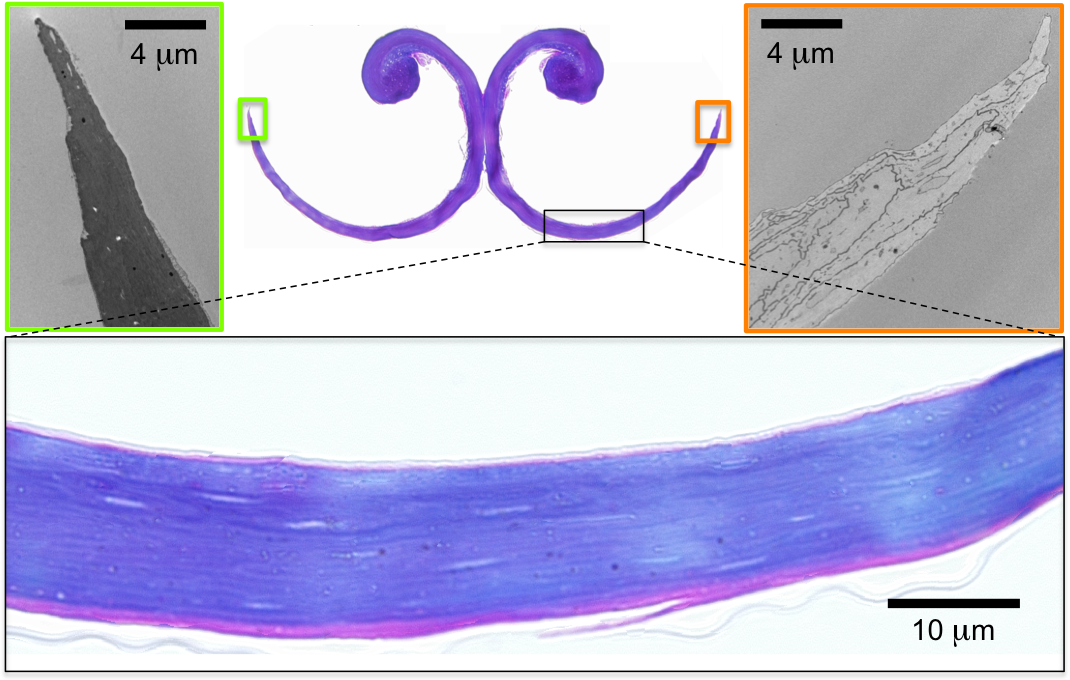


**Figure S2. Cross section close-ups of the outward halves of the groove walls, contrasting staining methods, in a** **Ruby-throated Hummingbird (*Archilochus colubris*) tongue.** At the top left, an electron micrograph stained with uranyl acetate and lead citrate. At the top right, an electron micrograph vapor-stained with RuO_4_. Bottom, light micrograph stained with methylene blue/azure II (counterstained with fuchsine). Note that the most outward halves of the groove wall in cross section are entirely composed of the cornified layer.


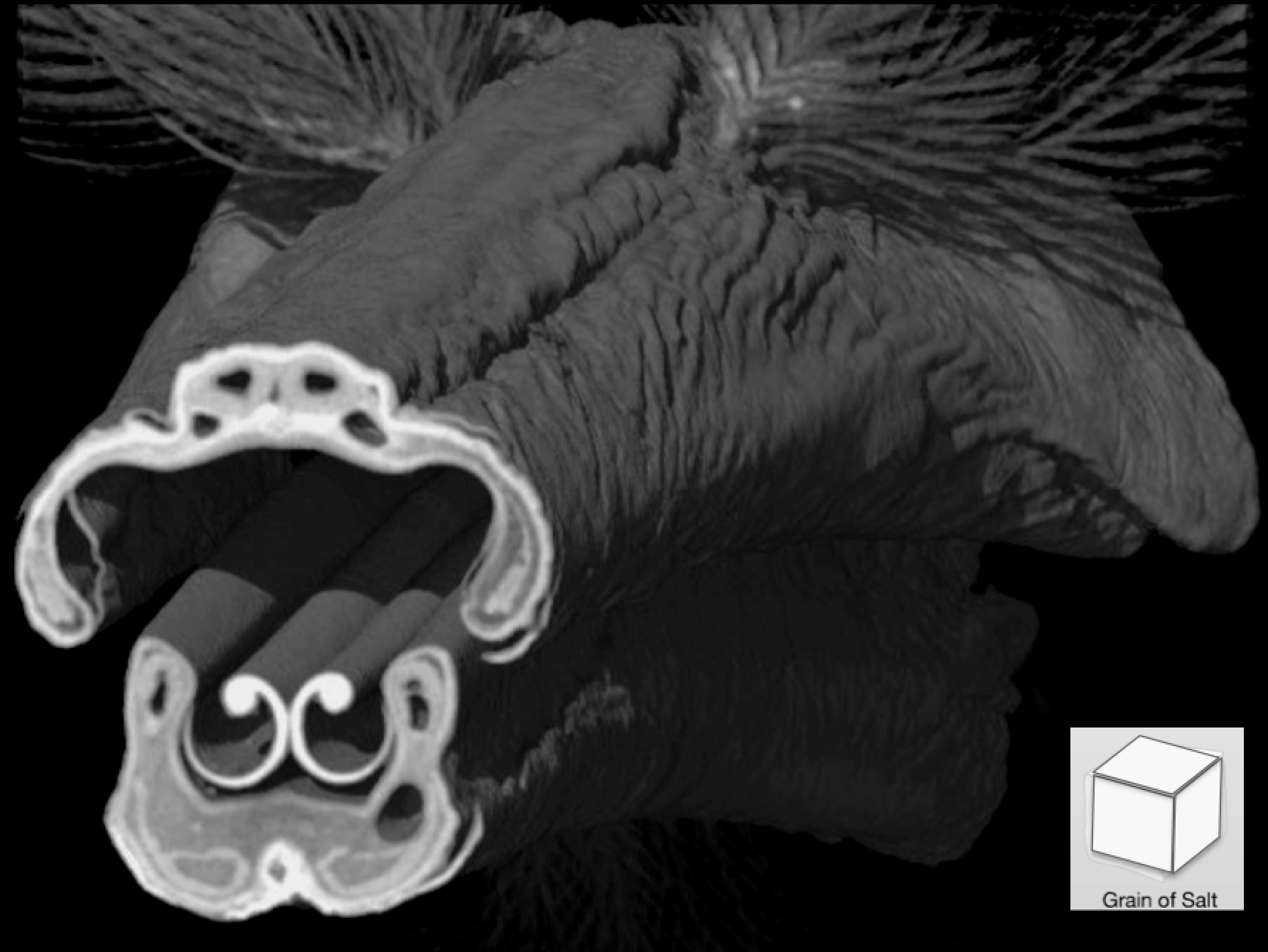


**Video S1. MicroCT rendering (rostro-cranial coronal cross sectioning) of the bill and tongue overlaying TEM micrographs; Ruby-throated Hummingbird (*Archilochus colubris*).** This virtual model of the internal three-dimensional architecture help us to understand the fit between bill and tongue. Additionally, merging microCT, light, and electron microscopy allows us to understand the key morphological features for the tongue functioning linking them across spatial scales. Known objects are placed at the same scale at the different zoom levels in order to contextualize the structures shown.

**
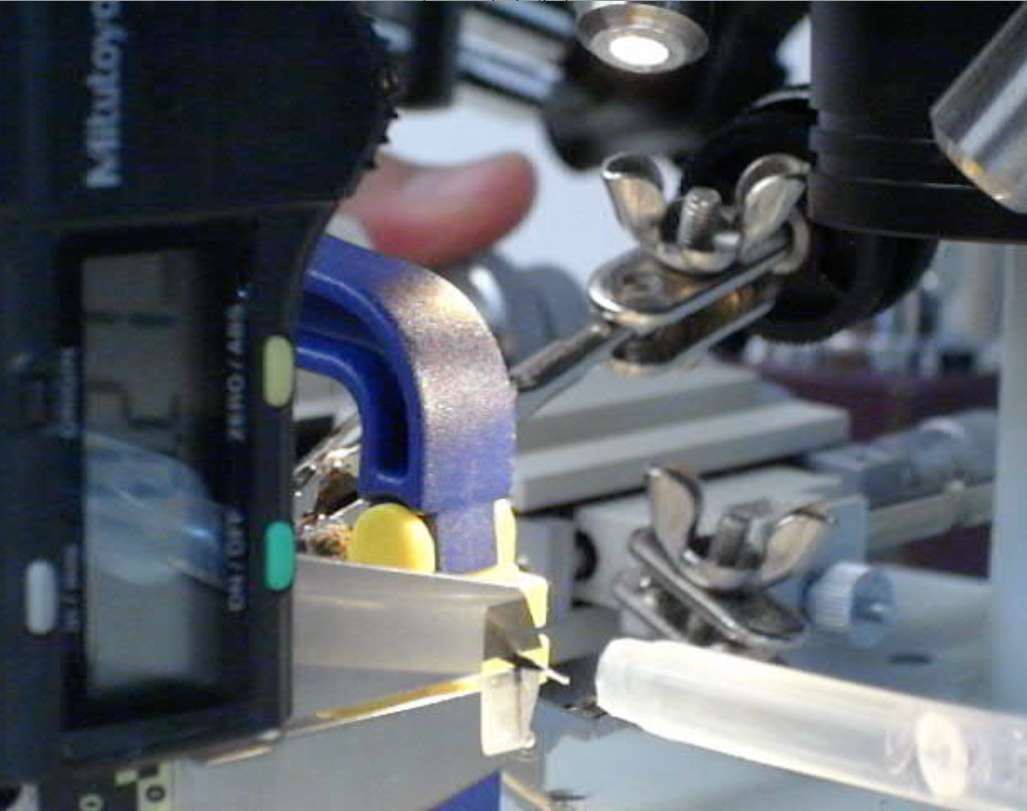
**

**Video S2.** **Control of the** **setup to film *post-mortem* tongue fluid interactions.** Under a dissecting microscope (*cf.* Fig S2), this control system emulates reciprocating tongue movements and allows for fine control of the distance to the nectar surface using micromanipulators. I coupled a high-speed camera to capture the details of tongue fluid interactions (e.g. Video S3).


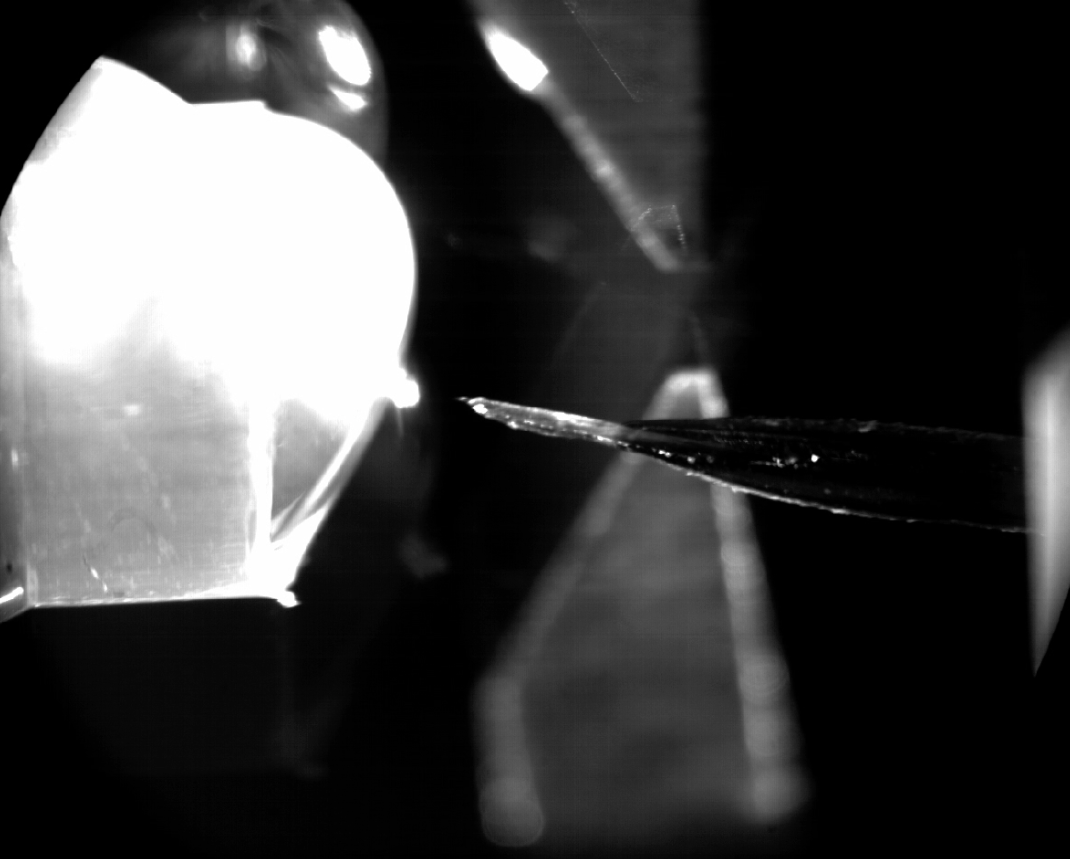


**Video S3.** **High-speed recording of *post-mortem* tongue expansive filling.** A slow motion (165 times slower than real time) video of the lateral view of a dissected Ruby-throated Hummingbird (*Archilochus colubris*) focusing on the bill tip – tongue – nectar interaction. The tongue protraction is controlled by micromanipulators (Video S2). Footage obtained under a dissecting microscope (*cf.* Fig S2) with a flat surface mirror to achieve the side view.


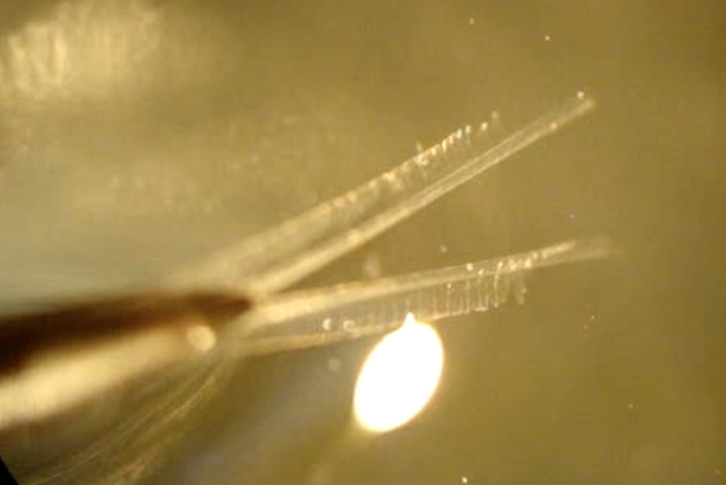


**Video S4.** **Recording of *post-mortem* tongue fluid trapping.** A real-time video of the dorsal view of a deceased Ruby-throated Hummingbird (*Archilochus colubris*) focusing on the bill tip – tongue – nectar interaction. Footage obtained under a dissecting microscope immersing the tongue in a nectar reservoir to appreciate the instantaneous change in shape when the tongue transitions form air (out of focus) to nectar (in focus) and *vice versa*.


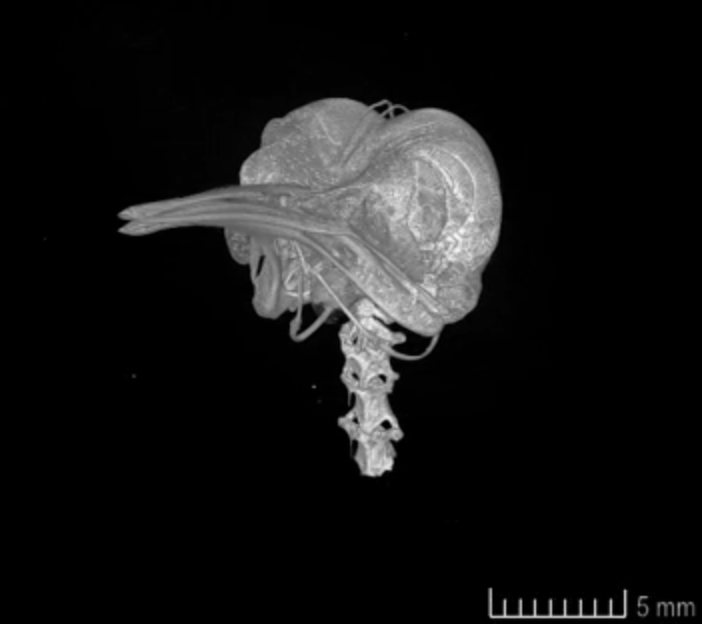


**Video S5.** **Three-dimensional digital rendering of a microCT scan of the skull of a Ruby-throated Hummingbird (*Archilochus colubris*).** This spinning reconstruction makes it possible to follow and visualize the elongated epibranchials surrounding the skull. In this case, the tongue is retracted inside the bill.
